# Prior antiviral treatment and mortality among patients with hepatitis C virus-related hepatocellular carcinoma: A national cohort study

**DOI:** 10.1371/journal.pone.0255624

**Published:** 2021-08-03

**Authors:** Dong Hyun Sinn, Danbee Kang, Yun Soo Hong, Kwang Cheol Koh, Eliseo Guallar, Juhee Cho, Geum-Youn Gwak

**Affiliations:** 1 Department of Medicine, Samsung Medical Center, Sungkyunkwan University School of Medicine, Seoul, South Korea; 2 Department of Clinical Research Design and Evaluation, SAIHST, Sungkyunkwan University, Seoul, South Korea; 3 Center for Clinical Epidemiology, Samsung Medical Center, Sungkyunkwan University, Seoul, South Korea; 4 Department of Epidemiology and Medicine and Welch Center for Prevention, Epidemiology and Clinical Research, Johns Hopkins Medical Institutions, Baltimore, MD, United States of America; University of Campania "Luigi Vanvitelli", ITALY

## Abstract

**Background:**

The current antiviral treatments available for hepatitis C virus (HCV) infection decrease the risk of hepatocellular carcinoma (HCC). Hence, patients with HCV infection who have not received antiviral treatment and have developed HCC may be those who missed timely antiviral treatment for HCV. However, the proportion of patients who missed timely antiviral treatment and its implications are largely unexplored.

**Methods:**

A nationwide retrospective cohort of 4,592 newly diagnosed HCV-related HCC patients (2013–2017) was identified from the Korean National Health Insurance Service database. Prior antiviral treatment for HCV was defined as a history of at least one HCV-specific antiviral treatment before HCC diagnosis. The outcome was all-cause mortality.

**Results:**

Prior antiviral treatment for HCV was identified in 802 (17.4%) patients, and 16%, 16%, 17%, 19%, and 19% of patients received antiviral treatment in the years 2013, 2014, 2015, 2016, and 2017, respectively (*P* = 0.21). During 8,085 person-years of follow-up (median, 1.4; maximum, 5.3 years of follow-up), 1,970 patients died. Mortality rates were lower in patients with prior antiviral treatment (15 deaths/100 person-years) than in those without prior antiviral treatment (27 deaths/100 person-years). The adjusted hazard ratio (95% confidence interval) for all-cause mortality on comparing patients who did and did not receive prior antiviral treatment was 0.68 (0.59, 0.79).

**Conclusion:**

Timely antiviral treatment for HCV was suboptimal at the population level. Prior antiviral treatment for HCV reduced mortality rate in HCV-related HCC patients. Intensive HCV control strategies are needed to reduce the number of patients with HCV infection who miss timely HCV treatment.

## Introduction

Hepatitis C virus (HCV) infection is a major risk factor for hepatocellular carcinoma (HCC) [1]. HCV infection causes acute hepatitis that can progress to chronic hepatitis, cirrhosis, and HCC [2]. HCV-related HCC is potentially preventable, and it is a rational target for cancer preventive interventions [3]. HCV infection can be prevented by blocking the routes of HCV transmission [4]. Effective and well-tolerated antiviral treatments for HCV are available [5]. Notably, the development of direct-acting antivirals (DAAs) for HCV has revolutionized patient care, with HCV eradication rates of over 90% [6, 7]. With the eradication of HCV through antiviral treatment, the risk of HCC diminishes, although it is not completely eliminated [8–10].

Ideally, all patients with HCV infection should be identified early before they develop HCC. However, chronic HCV infection remains clinically dormant for decades, and many people are unaware of the presence of HCV infection [11, 12]. Hence, many patients with HCV infection receive treatment after developing HCC and miss the opportunity for receiving timely antiviral treatment [5]. Patients without prior antiviral treatment can be regarded as a subgroup where earlier initiation of antiviral treatment would have provided significant benefits [5]. However, to date, there is limited information regarding the proportion of HCV-related HCC patients without prior antiviral treatment and its implications on clinical outcomes. In this study, we used a nationally representative sample to evaluate the proportion of prior antiviral treatment for HCV among newly diagnosed HCV-related HCC patients and its relationship with survival.

## Methods

### Study population and design

This is a population based retrospective cohort study using the Korean National Health Insurance Service (NHIS) database. We included men or women who were aged ≥ 40 years and were newly diagnosed with HCV-related HCC between January 1, 2013, and December 31, 2017 (N = 5,446). HCC was defined as the registration of the C code (International Classification of Diseases, 10th Revision [ICD-10]: code C22.0) more than three times within a year or an inpatient hospitalization with a C22.0 code. HCV infection was defined based on the registration of the ICD-10 code B18.2 before or within 6 months of HCC diagnosis.

**Fig 1:**
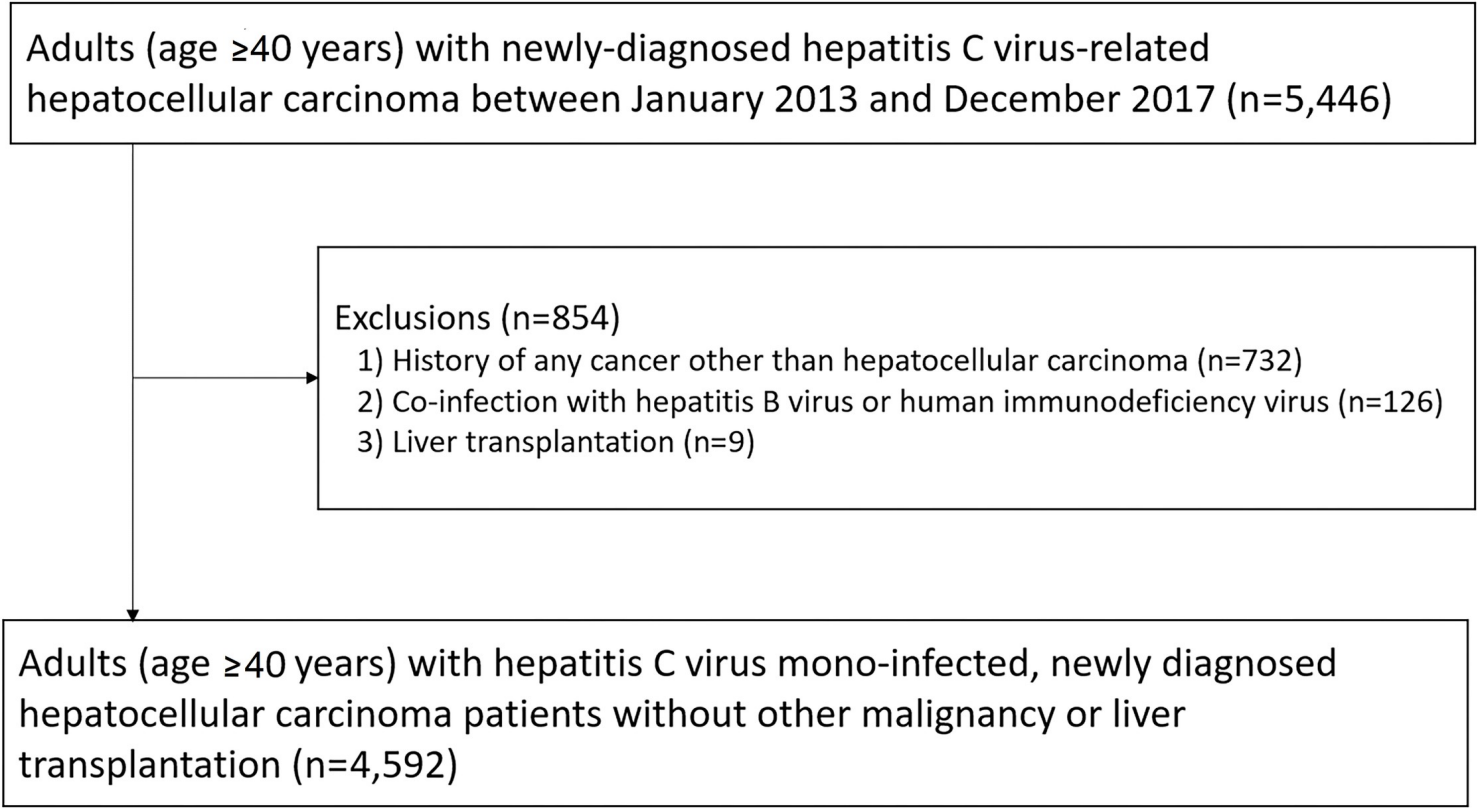
Flowchart of study participants.

Among them, we excluded patients with history of any cancer other than HCC (N = 732), co-infection with hepatitis B virus (ICD-10 code: B18.0, B18.1, and Z22.5) or human immunodeficiency virus (ICD-10 code: B20, B21, B22, and B24) (N = 126), and history of liver transplantation (ICD-10 code: Z94.4) before HCC diagnosis (N = 9). The final sample size was 4,592 (2,974 men and 1,618 women) (Fig 1). The Institutional Review Board of the Samsung Medical Center approved this study and waived the requirement for informed consent because of the retrospective nature of our study using de-identified data.

### Data sources

Korea has a single payer system that all population are covered by NHIS. Costs for medical service are reimbursed if medical service for hospital visits, procedures, and prescriptions are approved medical service by NHIS. We used the NHIS database provided by the Korean NHIS [13], which is a government-affiliated agency under the Korean Ministry of Health and Welfare that supervises all medical services in Korea. The NHIS maintains records insurance eligibility, age, sex, income level, and all of the insurance covered inpatient and outpatient visits, procedures, and prescriptions using the ICD-10 and the Korean Drug and Anatomical Therapeutic Chemical Codes [14, 15]. During the study period, medical costs for inpatients and outpatients visit, procedures were covered by NHIS. Cost for interferon-based treatments and DAAs were approved and reimbursed by NHIS at early 1990s and 2015, respectively. The vital status (reference date: December 31, 2017) was ascertained from mortality and population registration data from Statistics Korea.

### Study variables

The main exposure was antiviral treatment for HCV before HCC diagnosis. Antiviral treatment for HCV was defined as a history of at least one HCV-specific treatment code (S1 Table). The evaluated treatments included interferon, peginterferon, ribavirin, or DAAs.

Initial treatment of HCC, including surgical resection, liver transplantation, radiofrequency ablation, transarterial chemoembolization, radiation therapy, and systemic chemotherapy, was identified based on the claim codes. Treatment was categorized into curative intent treatment (surgical resection, liver transplantation, and radiofrequency ablation) and palliative treatment (transarterial chemoembolization, radiation therapy, and systemic chemotherapy). Comorbidities during the year prior to cancer diagnosis were defined using ICD-10 codes [14] and summarized using the Charlson comorbidity index (CCI) [16, 17]. CCI gives score of 1 for mild case, and score of 3 for moderate to severe disease. Moderate to severe liver disease was defined when there was portal hypertension or related complications, including chronic hepatic failure (K72.1), hepatic failure (K72.9), portal hypertension (K76.6), hepato-renal syndrome (K76.7), esophageal varix with bleeding (I85.0), esophageal varix without bleeding (I85.9), gastric varices (I86.4), and esophageal varices without bleeding in diseases classified elsewhere (I98.2). Information regarding demographics, income levels, and residential area was based on claim codes in the insurance eligibility database.

### Statistical analysis

The outcome of the study was all-cause mortality. Person-time was calculated from the date of HCC diagnosis to the date of death or the end of the study period (December 31, 2017). Survival curves were generated by the Kaplan–Meier product-limit method and compared by log-rank tests. We calculated hazard ratios (HRs) with 95% confidence intervals (CIs) for all-cause mortality using Cox proportional hazards regression models. To account for potential confounding factors at the time of HCC diagnosis, we adjusted for sex, age, year of HCC diagnosis, income level category (≤30^th^ percentile, >30^th^-<70^th^ percentile, and >70^th^ percentile), residential area (metropolitan area, including Seoul, six metropolitan cities, 15 cities with a population of >500,000 people, and those officially designated as municipal cities (http://www.mois.go.kr), or rural area), and initial treatment. Since patient survival could be clustered by hospital, we used “hospital of HCC diagnosis” as a stratification factor in the Cox models. We examined the proportional hazards assumption using plots of the log (-log) survival function and Schoenfeld residuals.

We performed subgroup analyses to evaluate the association of antiviral treatment with all-cause mortality by subgroups defined a priori by age (<65 years vs. ≥65 years), sex (male vs. female), income percentile (<30^th^ vs. 30^th^-<70^th^ vs. ≥70^th^), CCI score (0–1 vs. ≥2), and initial treatment allocation (curative vs. palliative or no treatment).

In addition, we used logistic regression to identify the factors associated with HCV treatment before HCC. A *P*-value < .05 was considered statistically significant. All analyses were performed using STATA version 14 (StataCorp LP, College Station, TX, USA).

## Results

**Fig 2:**
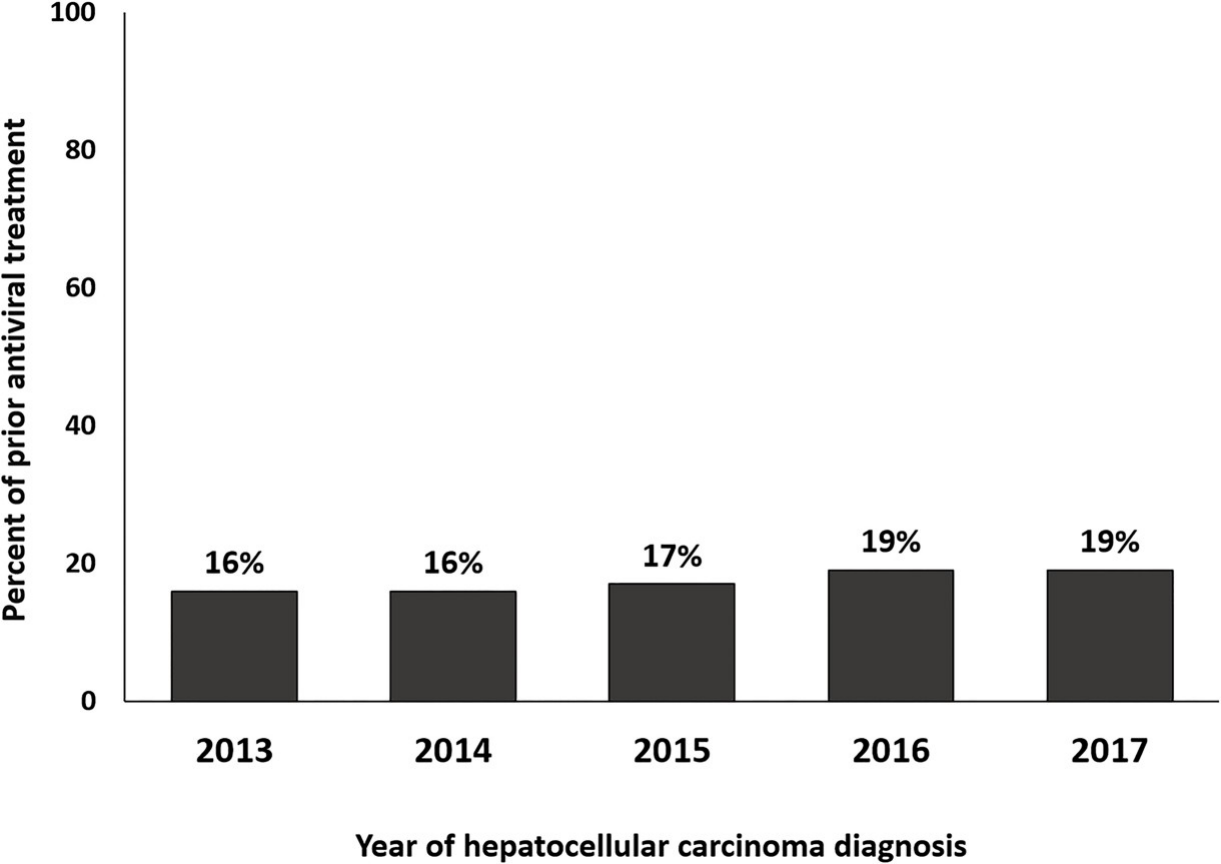
Proportions of patients with hepatitis C virus-related hepatocellular carcinoma in groups with and without prior hepatitis C virus treatment by year of diagnosis.

Between 2013 and 2017, 4,592 patients were newly diagnosed with HCV-related HCC, and 802 (17.4%) patients had received antiviral treatment for HCV before the HCC diagnosis. Among 3,790 patients without HCV treatment before HCC diagnosis, 80.3% were diagnosed with HCV infection at least 15 months before HCC diagnosis (S2 Table). Between 2013 and 2017, the proportion of patients who had received antiviral treatment before HCC diagnosis increased gradually, but the difference was not significant (*P* = 0.21) (Fig 2). Those who received prior antiviral treatment were younger and received curative therapies for HCC more commonly than patients who did not receive prior antiviral treatment (Table 1). Income percentile and residential area were not different between patients who did and did not receive prior antiviral treatment.

**Table 1:** Characteristics of study participants with and without prior hepatitis C virus treatment who were diagnosed with hepatocellular carcinoma.

	Without HCV treatment before HCC diagnosis	With HCV treatment before HCC diagnosis	*P* value
	*N* (%)	*N* (%)	
**Number of patients**	3,790	802	
**Sex**			0.11
Male	2,435 (64.2)	539 (67.2)	
Female	1,355 (35.8)	263 (32.8)	
**Age (years)**	68.6 (10.6)	63.0 (8.9)	<0.01
**Income percentile**			0.2
≤30^th^	1,175 (31.0)	272 (33.9)	
>30^th^-≤70^th^	1,121 (29.6)	217 (27.1)	
>70^th^	1,494 (39.4)	313 (39)	
**Residential area**			0.15
Metropolitan	2,370 (62.5)	523 (65.2)	
Rural	1,420 (37.5)	279 (34.8)	
**Initial treatment**			<0.01
No treatment	1,537 (40.6)	276 (34.4)	
Resection	531 (14.0)	139 (17.3)	
RFA	484 (12.8)	136 (17)	
TACE	976 (25.8)	196 (24.4)	
Targeted therapy	59 (1.6)	8 (1.0)	
Others	203 (5.4)	47 (5.9)	
**Charlson comorbidity index**	2 (1–3)	2 (1–4)	0.34

The income percentile is based on household income.

Abbreviations: HCV, Hepatitis C virus; HCC, Hepatocellular carcinoma; RFA, Radiofrequency ablation; TACE, Transarterial chemoembolization.

**Fig 3:**
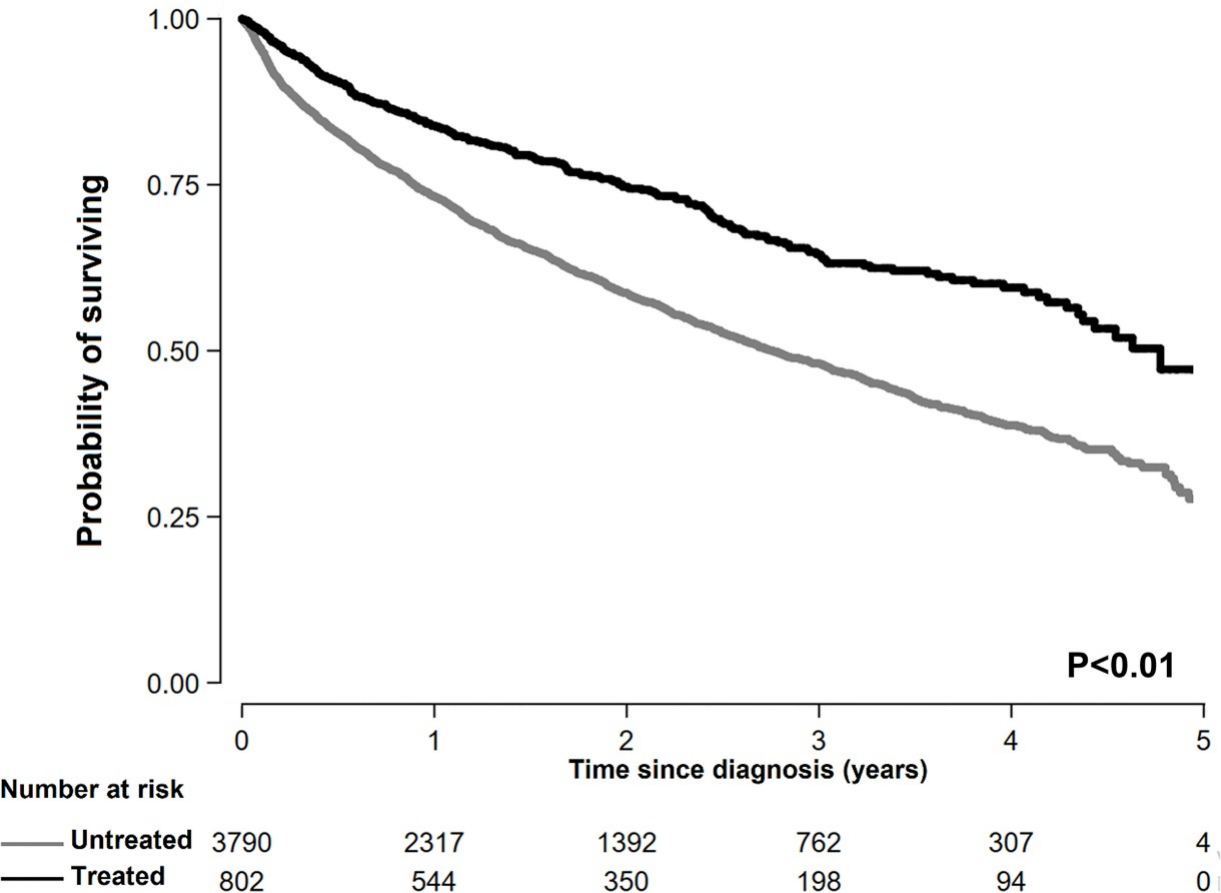
Kaplan–Meier survival curves of patients with hepatitis C virus -related hepatocellular carcinoma according to prior hepatitis C virus treatment.

**Fig 4:**
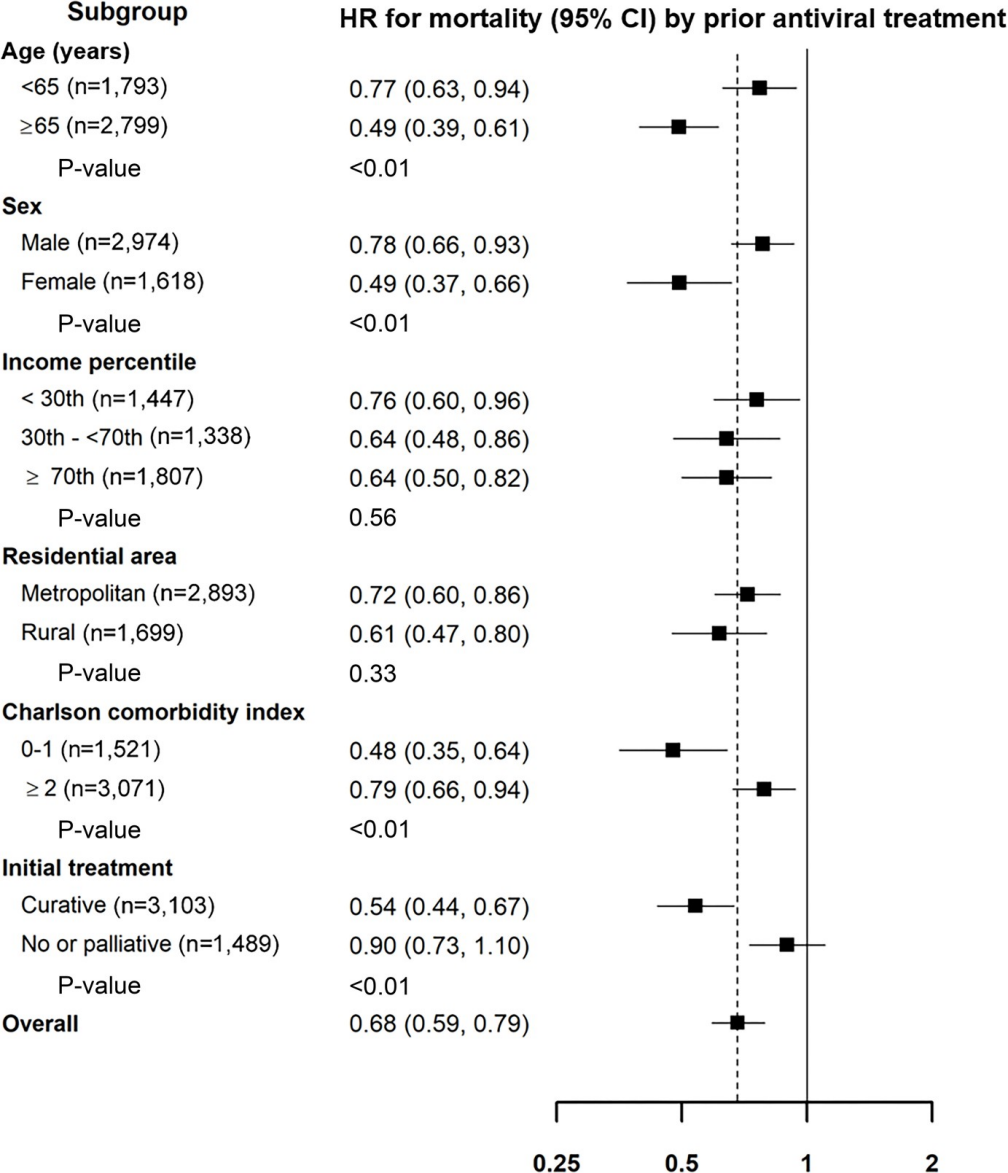
Hazard ratios (95% confidence intervals) for all-cause mortality of patients with hepatitis C virus-related hepatocellular carcinoma according to prior hepatitis C virus treatment in selected subgroups. Adjusted for sex, age, year of hepatocellular carcinoma diagnosis, income percentile (≤30^th^, >30^th^-≤70^th^, and >70^th^), residential area (metropolitan or rural), initial treatment (curative or palliative), and Charlson comorbidity index. (HR, hazard ratio; CI, confidence interval).

During 8,085 person-years of follow-up (median, 1.4; maximum, 5.3 years of follow-up), 1,970 patients died (1,738 and 232 patients with and without antiviral treatment, respectively). Mortality rates were lower in patients with prior antiviral treatment (15 deaths/100 person-years) than in those without prior antiviral treatment (27 deaths/100 person-years) (Fig 3 and Table 2). After adjusting for sex, age, year of HCC diagnosis, income percentile (≤30^th^, >30^th^–≤70^th^, and >70^th^), residential area (metropolitan or rural), initial treatment (curative or palliative), and CCI, the HR (95% CI) for all-cause mortality when comparing patients with and without prior antiviral treatment was 0.68 (0.59, 0.79) (Table 2). The association between prior antiviral treatment and decreased all-cause mortality was observed in all the subgroups analyzed (Fig 4); however, the association was stronger for the factors of age ≥65 years, female sex, CCI 0–1, and curative treatment as compared to age <65 years, male sex, CCI score ≥2, and no treatment or palliative treatment.

**Table 2:** Hazard ratios (95% confidence intervals) for all-cause mortality of patients with hepatitis C virus-related hepatocellular carcinoma according to prior hepatitis C virus treatment.

	Person-years	No. of deaths	Incidence rate (per 100 person-years)	Crude HR (95% CI)	Adjusted HR (95% CI)
**HCV treatment before HCC diagnosis**					
No	6,520	1,738	27	*Reference*	*Reference*
Yes	1,565	232	15	0.58 (0.50, 0.68)	0.68 (0.59, 0.79)
*P-value*				<0.01	<0.01

Adjusted for sex, age, year of HCC diagnosis, income percentile (≤30^th^, >30^th^–≤70^th^, and >70^th^), residential area (metropolitan or rural), initial treatment (curative or palliative), and Charlson comorbidity index.

Abbreviations: HR, Hazard ratio; CI, Confidence interval: HCV, Hepatitis C virus; HCC, Hepatocellular carcinoma.

## Discussion

In this nationwide population-based study, only 17.4% of newly diagnosed HCV-related HCC patients had received prior antiviral treatment for HCV. This indicates that a large proportion of HCV-related HCC patients missed the opportunity for antiviral treatment for HCV before developing HCC. In addition, from 2013 to 2017, the proportion of patients who received prior antiviral treatment did not increase significantly (from 16.4% to 18.6%; *P* = 0.21). The low proportion of HCV-related HCC patients who received prior antiviral treatment suggests that a large proportion of patients with HCV infection missed the benefit of earlier antiviral treatment.

In this study, prior antiviral treatment was associated with better survival in HCV-related HCC patients. Prior antiviral treatment was an independent factor associated with outcome after adjusting for sex, age, year of HCC diagnosis, income percentile, residential area, initial treatment, and Charlson comorbidity index. The association between prior antiviral treatment and decreased all-cause mortality was consistently observed in all the subgroups analyzed. There are several prognostic factors of survival in HCV-related HCC patients, including age, sex, liver function, tumor stage, and treatment [18]. As the NHIS database does not have information regarding liver function and tumor stage, we cannot exclude the possibility that better survival of HCV-related HCC patients with prior antiviral treatment might be associated with better liver function and earlier tumor stage. However, it is also well known that HCV viremia is associated with poor outcomes and that antiviral treatment for HCV can reduce the risk of all-cause mortality and liver-specific mortality [19, 20]. Antiviral treatment can eradicate HCV; the reported cure rate with antiviral treatment is over 90% with DAAs and about 40–80% with peginterferon and ribavirin treatment [21, 22]. NHIS database has information on antiviral treatment but not on the outcomes of antiviral treatment. However, we expect that HCV infection would be cured in a significant proportion of patients who received prior antiviral treatment. HCV eradication in patients with HCV infection reduces a patient’s risk of cirrhosis and can reduce mortality [23]. Prior antiviral treatment can result in better preservation of liver function and prevent HCC recurrence, thereby improving the outcomes of HCV-related HCC patients compared to those of patients without prior antiviral treatment [23–25].

Due to the nature of NHIS data, we were not able to identify the exact reason for not receiving antiviral treatment before HCC diagnosis (e.g., unawareness of HCV infection, awareness of infection but no treatment due to comorbidities, and cost issues). In a nationwide telephonic interview involving 1,003 participants from the general population of South Korea, the self-reported testing rate for HCV was less than 10% [12]. In a cancer screening cohort study, only 34.9% of HCV carriers were aware of their infection status [26]. In a nationwide multicenter study, wherein linkage to care was analyzed, 72.8% patients received antiviral treatment when HCV viremia was identified [27]. In a nationwide telephonic interview, the treatment rate was 80% among people who reported positive results for HCV [12]. In our study, among patients without HCV treatment before HCC diagnosis, over 80% of the HCC patients had been diagnosed with HCV infection more than 1 year before HCC diagnosis. This indicates that many of HCV-infected patients would know their HCV infection status but did not receive antiviral treatment for unknown reasons. However, people might not aware their HCV infection status even if they had tested for it. Further studies are required to see the exact reason behind low antiviral treatment rate before HCC diagnosis. In addition, public health campaign to increase awareness and screening for HCV infection would be necessary to decrease the proportion of HCV-related HCC patients.

There were several limitations in this study. First, these data were based on administrative claims for reimbursement, and it did not include information on important factors that could potentially affect the survival of HCC patients, such as tumor stage and smoking status. In addition, we did not have information on alcohol liver disease or hepatic steatosis which can affect worse clinical outcomes [28, 29]. Second, severity of liver disease was assessed using ICD code and classified according to CCI [16, 17]. This can be less accurate than using other severity scores specifically designated to liver disease severity such as Child-Pugh score or model for end-stage liver disease score. However, CCI is a valid measure which is widely used in studies using administrative data to control for the overall burden of comorbidities [30]. Third, we were not able to determine HCV treatment response from the database which would be associated with outcomes. While we identified patients treated with DAAs which was known for high success rate, we were not able to conduct further analysis due to small number of DAA-treated patients. The approval for DAAs in South Korea was obtained in 2015, and only 11 patients in our study was treated with DAAs. Fourth, engagement in care and receiving regular HCC surveillance can also affect patient outcome in HCV-infected patients [31, 32]. It is possible that those who received antiviral treatment were more likely to engage in care and receiving HCC surveillance. They would be more likely to be diagnosed at early-stage HCC, and had improved survival [33]. However, due to the nature of the claim data, we were not able to include information on HCC surveillance.

To summarize, we observed that the current status of HCV control is suboptimal at the population level, indicated by a very low percentage of HCV-related HCC patients who received prior antiviral treatment. HCV-related HCC patients without prior antiviral treatment had 30% higher mortality compared to those with prior antiviral treatment. Our analysis justifies the need for a more intensive HCV screening and treatment strategy to reduce HCV-related HCC burden and suggests that prior antiviral treatment among newly diagnosed HCV-related HCC patients can be a surrogate for measuring the effectiveness of an HCV control strategy in a given area.

## Supporting information

S1 Table List of therapeutic agents for hepatitis C virus infection. (DOCX)

S2 Table Interval between diagnosis of hepatis C virus infection and hepatocellular carcinoma. (DOCX)
