# Supplementary material for: Prior antiviral treatment and mortality among patients with hepatitis C virus-related hepatocellular carcinoma: A national cohort study
Source: PLoS One. 2021 Aug 3;16(8):e0255624. doi: 10.1371/journal.pone.0255624 (PMC8330890; doi:10.1371/journal.pone.0255624)
Supplement: S1 Table — (DOCX) [file pone.0255624.s001.docx]

**S1 Table. List of therapeutic agents for hepatitis C virus infection.**

| **Treatment** | **Classification** | **Treatment code** |
| --- | --- | --- |
| Interferon alfa-2a | Interferon-based | 175502BIJ, 175504BIJ, 175530BIJ, 175503BIJ, 175506BIJ |
| Interferon alfa-2b | Interferon-based | 175630BIJ, 175631BIJ, 175606BIJ, 175602BIJ, 175609BIJ |
| Peginterferon alfa-2a | Interferon-based | 452601BIJ, 452602BIJ, 452630BIJ |
| Peginterferon alfa-2b | Interferon-based | 454832BIJ, 454833BIJ, 454835BIJ, 454830BIJ, 454831BIJ, 454834BIJ |
| Ribavirin |  | 223601ACH, 23604ACH |
| Boceprevir | DAA | 627801ACH |
| Asunaprevir | DAA | 638001ACH |
| Daclatasvir | DAA | 638101ATB |
| Sofosbuvir | DAA | 644401ATB |
| Ledipasvir | DAA | 645800ATB |
| Elbasvir/grazoprevir | DAA | 657000ATB |
| Ombitasvir/paritaprevir/ritonavir | DAA | 658600ATB |
| Dasabuvir | DAA | 658701ATB, 638101ATB |
| Glecaprevir/pibrentasvir | DAA | 669700ATB |

Abbreviations: DAA, Direct antiviral agent.
