# Supplementary material for: Prior antiviral treatment and mortality among patients with hepatitis C virus-related hepatocellular carcinoma: A national cohort study
Source: PLoS One. 2021 Aug 3;16(8):e0255624. doi: 10.1371/journal.pone.0255624 (PMC8330890; doi:10.1371/journal.pone.0255624)
Supplement: S2 Table — (DOCX) [file pone.0255624.s002.docx]

**S2 Table. Interval between diagnosis of hepatis C virus infection and hepatocellular carcinoma**

| **Diagnosis of HCV infection** | **Without HCV treatment before HCC diagnosis**  ***N* (%)** |
| --- | --- |
| after HCC diagnosis | 219 (5.8) |
| < 3 months before HCC diagnosis | 317 (8.4) |
| 3-15 months before HCC diagnosis | 211 (5.6) |
| > 15 months before HCC diagnosis | 3,043 (80.3) |

Abbreviations: HCV, hepatitis C virus; HCC, hepatocellular carcionma
